# Using Self-Determination Theory to Examine Musical Participation and Well-Being

**DOI:** 10.3389/fpsyg.2019.00405

**Published:** 2019-03-01

**Authors:** Amanda E. Krause, Adrian C. North, Jane W. Davidson

**Affiliations:** ^1^The Melbourne Conservatorium of Music, The University of Melbourne, Parkville, VIC, Australia; ^2^School of Psychology, Curtin University, Perth, WA, Australia

**Keywords:** musical participation, well-being, Self-Determination Theory, psychological needs, autonomous motivation

## Abstract

A recent surge of research has begun to examine music participation and well-being; however, a particular challenge with this work concerns theorizing around the associated well-being benefits of musical participation. Thus, the current research used Self-Determination Theory to consider the potential associations between basic psychological needs (competence, relatedness, and autonomy), self-determined autonomous motivation, and the perceived benefits to well-being controlling for demographic variables and the musical activity parameters. A sample of 192 Australian residents (17–85, *M*_age_ = 36.95), who were currently participating in a musical activity at the time, completed an online questionnaire. Results indicated that females were more likely to perceive benefits to their well-being; and that how important an individual considers music in their life was positively related to perceived well-being. Importantly, the analyses also revealed that the basic needs of competency and relatedness were related to overall perceived well-being as well as specifically social, cognitive, and esteem dimensions of well-being. Autonomous motivation demonstrated significant associations with both an overall well-being score as well as four of five specific well-being subscales measured. Collectively, the findings indicate that Self-Determination Theory offers a useful theoretical framework to understanding the relationship between musical participation and well-being. Further, the pattern of findings reiterates the positive associations between musical participation and one’s psychosocial well-being, with broad implications for people involved in the facilitation of musical activity.

## Introduction

There is a growing interest in researching the possible relationship between music and well-being (Clift et al., 2008; Skingley et al., 2011; MacDonald et al., 2012; MacDonald, 2013). However, research on the subject faces a number of challenges. One, in particular, concerns the challenge of producing systematic, empirical evidence to support claims that are sometimes taken to be self-evident (Skingley et al., 2011). Relatedly, much of the relevant work can be challenged on the grounds of a lack of theoretical grounding (Clift and Hancox, 2010). Recently, researchers (e.g., Küpers et al., 2014; Evans, 2015; Krause and Davidson, 2018) have suggested the utility of Self-Determination Theory as a framework to consider musical participation and well-being. Self-Determination Theory has been applied to a wide range of social psychological behaviors, spanning health, education, and social relationships, and is supported by a growing body of research (Evans, 2015). Indeed, Self-Determination Theory has been used to explain a range of behaviors that involve motivation over extended periods of time (e.g., Georgiadis et al., 2006; Reinboth and Duda, 2006; Sheldon and Krieger, 2007; Alivernini and Lucidi, 2011; Jang et al., 2012). Therefore, the present research examined perceived well-being associated with active musical participation using Self-Determination Theory as a theoretical framework.

### Self-Determination Theory

Self-Determination Theory is concerned with human motivation, development, and wellness: it outlines how the concept of motivation relates to individuals’ affect, behavior, and well-being (Deci and Ryan, 2000, 2008). Self-Determination Theory is a macro theory, encompassing several mini-theories. Two of these theories, Basic Psychological Needs and Organismic Integration Theory, are particularly relevant to musical participation and its perceived well-being benefits. Self-Determination Theory argues that internal, external, and contextual factors, combine to influence the fulfillment of needs by either increasing or decreasing one’s motivation to participate (Ryan and Deci, 2000). In this way, Self-Determination Theory can be used to understand how engagement, such as musical participation, can be fostered. Importantly, motivation, an important element to starting and continuing in musical activities (O’Neill and McPherson, 2002; McPherson and O’Neill, 2016), is central to Self-Determination Theory, and both Basic Psychological Needs and Organismic Integration Theory in particular. In this context we note that participation in music, for the majority, takes place during leisure time and/or or an elective basis: given that Self-Determination Theory has been used to study ongoing engagement with other leisure and elective activities, it is a suitable candidate for explaining ongoing engagement with music.

Basic psychological needs theory states that people strive to satisfy three innate needs, namely competence, relatedness, and autonomy (Ryan and Deci, 2002; Hagger et al., 2006). Competence refers to the need to be effective in one’s efforts; relatedness concerns being connected socially, and integrated into a social group; and autonomy concerns the need to feel that one’s pursuits are self-governed and self-endorsed (Ryan and Deci, 2002). Deci and Ryan assert that needs for competence, relatedness, and autonomy are universal – they are essential, regardless of culture and life domain (Deci and Ryan, 1985b, 2000; Ryan and Deci, 2002). Both internal, personal factors and the social environment influence the degree to which the three needs are met (Ryan and Deci, 2000; Quested et al., 2018). Meeting these needs leads to personal growth, vitality, and well-being (Deci and Ryan, 2000; Ryan and Deci, 2002).

Organismic integration theory (Deci and Ryan, 1985a, 1991; Ryan and Deci, 2000) differentiates types of motivation (Ryan and Connell, 1989; McLachlan et al., 2011). It places particular emphasis on the quality of motivation, rather than merely the quantity (Niven and Markland, 2016). Six different types of motivation exist, and are often conceptualized as lying on a continuum (Gagné and Deci, 2005; McLachlan et al., 2011; Wilson et al., 2012; MacIntyre et al., 2018). Intrinsic motivation lies at the internal end of this continuum, and represents self-determined, internalized motivation. External motivation lies at the other end of the continuum, and refers to motivation that is characterized by engagement for reasons completely external to oneself. Three additional types of extrinsic behavioral regulation which differ in terms of the degree to which the motivation is internalized are positioned between these two poles. The three different types of external motivation are termed integrated, identified, and introjected (McLachlan et al., 2011). Integrated regulation is the most autonomous form of extrinsic regulation, followed by identified, and introjected (the least autonomous form of extrinsic motivation). Integrated regulation concerns behavior that is fully assimilated and consistent with one’s self; identified regulation refers to behavior based on attaining “personally valued outcomes rather than for enjoyment or interest”; and introjected regulation concerns enhancing self-worth by avoiding negative affective states, such as shame and guilt (McLachlan et al., 2011, p. 724; Wilson et al., 2012). Amotivation refers to the absence of clear motivation or intentions (Markland and Tobin, 2004; McLachlan et al., 2011). The theory recognizes that people’s actions within a given domain are simultaneously the product of several different motivations along the continuum; these can be accounted for by calculating a relative autonomy index (RAI). This index score, for which a higher score indicates greater autonomy, serves as an aggregate representation of how intrinsically or extrinsically motivated someone is Seymour and Peterman (2018). Organismic integration theory states that the fulfillment of psychological needs will lead to internalized motivation (Gagné and Deci, 2005).

The fulfillment of the basic psychological needs is fostered in environments that are autonomy-supportive and hindered in environments that are controlling (Bonneville-Roussy et al., 2013). Clear evidence of this comes from previous education research using Self-Determination Theory. Studies have found that students are more engaged and persistent in autonomy supportive activities and environments (e.g., Reeve et al., 2004; Hagger et al., 2015). In contrast, students suffer in environments in which teachers are more controlling (e.g., Soenens et al., 2012; Bonneville-Roussy et al., 2013). In short, in educational settings, autonomy support is associated with students having more self-determined forms of motivation and higher perceptions of competence (e.g., Williams and Deci, 1998) as well as higher levels of enjoyment, engagement, performance, and persistence (e.g., Black and Deci, 2000; Vansteenkiste et al., 2004; Chatzisarantis and Hagger, 2009; Niemiec and Ryan, 2009; Jang et al., 2010; Bonneville-Roussy et al., 2013; Oga-Baldwin et al., 2017; Ulstad et al., 2018).

Another claim of Self-Determination Theory is that satisfaction of needs is associated with well-being (Deci and Ryan, 2000; Milyavskaya and Koestner, 2011; Lombas and Esteban, 2018). Research has shown this in work settings (e.g., Ilardi et al., 1993; Deci et al., 2001; Baard et al., 2004; Van den Broeck et al., 2016) and healthcare settings, such as aged-care, where an autonomy-supportive environment has been associated with better well-being (Deci and Ryan, 1987; Ferrand et al., 2014). Research also links satisfying needs to perceived well-being with regard to leisure pursuits (Coleman and Iso-Ahola, 1993), including predominantly exercise (e.g., Chatzisarantis and Hagger, 2009; Lovell et al., 2016; Niven and Markland, 2016; Sebire et al., 2016), but also other pursuits such as community gardening (e.g, Quested et al., 2018) and relationship functioning (e.g., Patrick et al., 2007). As Coleman and Iso-Ahola (1993) found, leisure activities which promote fulfilling participants’ basic needs, therefore, promote self-determination and are beneficial to well-being (see also Deci and Ryan, 2000; Ferrand et al., 2014). Indeed, Kuykendall et al. (2015) recent meta-analysis provided strong evidence that leisure engagement is consistently associated with subjective well-being as well as evidence that leisure satisfaction mediates the relationship between leisure engagement and well-being. It is therefore surprising that little research has considered musical participation and well-being using self-determination theory as a theoretical framework, given the prevalence of music participation as a leisure time activity (Laukka, 2007).

### Self-Determination Theory in Music Research

Self-determination theory has recently been used in work concerning both music education and music therapy (Douglas, 2011; Evans and Bonneville-Roussy, 2016; Lee et al., 2016; Valenzuela et al., 2018). However, as Evans (2015, p. 7) referenced in his conceptual overview concerning how self-determination theory might be used to consider motivation in music education, there have only been “a small number of studies.” In particular, music education researchers have focused on practice, both at the university and conservatoire level (Evans and Bonneville-Roussy, 2016; Valenzuela et al., 2018) and middle-school level (Schatt, 2018). Findings indicate that the fulfillment of psychological needs and autonomous motivation were associated with practicing more frequently and a higher quality of practice (Evans and Bonneville-Roussy, 2016). However, Schatt’s (2018) findings indicated differences by instrument and grade level with regard to levels of self-determination to practice, suggesting that personal and contextual factors pertaining to the musical activity can influence one’s motivation. Moreover, feelings of autonomy and competence are linked to intrinsic motivation and the experience of flow (Valenzuela et al., 2018) in practice; and autonomy support is also related to passion and persistence in music education (Bonneville-Roussy et al., 2013). Additional work has considered musical play at recess (Countryman, 2014) and singing games in and out of the classroom (Roberts, 2018), providing further support for applying self-determination theory to understand musical behaviors.

Given that motivation, broadly, has been recognized as an important feature with regard to both starting and continuing to participate in musical activities (O’Neill and McPherson, 2002; McPherson and O’Neill, 2016), researchers have also used basic psychological needs to consider who plays versus ceases playing (Evans et al., 2013; Freer and Evans, 2018). Evidence suggests that when the three basic psychological needs are met, people are more likely to continue participating in musical activities (Douglas, 2011; Evans et al., 2013). Findings concerning community band participation pointed to associations with autonomy and competence, specifically (Douglas, 2011). Indeed, students are more likely to continue participating in music as an elective subject when their psychological needs are met (Freer and Evans, 2018).

Further, importantly, research findings support positive associations between feelings of subjective well-being and the three psychological needs in the context of musical participation (Creech et al., 2013b). In Creech et al.’s (2013b, p. 40) study, “subjective well-being was found to be underpinned by a sense of purpose, feeling in control and autonomous, and receiving affirmation through positive social relationships that accord individuals with respect and status.” Autonomous motivation was also related to coping strategies by university music students (Bonneville-Roussy et al., 2017).

### Present Research

The current study aimed to examine musical participation and well-being relative to self-determination theory. In doing so, it addressed particular limitations of past research in this area. Firstly, while researchers, such as Evans (2015), have provided conceptual overviews applying self-determination theory to musical behaviors, “there is the need to test the ideas empirically” (MacIntyre et al., 2018, p. 702). Moreover, while limited prior research has considered self-determination theory and motivation within a musical context, very little of this work has considered the associated well-being benefits that might follow. Although the previous research on well-being and musical participation has demonstrated many perceived well-being benefits, the Krause et al. (2018) review highlighted the need to systematically and comprehensively consider these. In response to identifying a small number of broad categories to which these benefits align (such as social, emotional, and cognitive), Krause et al.’s measure was designed to measure perceived well-being holistically and employed in the present study.

Secondly, much of the previous work has been limited in scope and/or size by focusing on a particular, specific well-being benefit or a tightly-defined sample of participants (Krause et al., 2018). Therefore, the present study considered adult musical participation more broadly. It was not limited to only formal music education settings or to university and conservatory students; rather in spirit of life-long engagement, the present study considers musical participation in various contexts among a community sample. In turn, the present analyses included demographic variables and the context of the musical activity as potential covariates within the analyses.

Consequently, the present study aimed to identify associations between psychological needs, motivation, and well-being in the context of musical participation. In line with past research, it was hypothesized that the three basic psychological needs outlined by self-determination theory (autonomy, competence, and relatedness) would be positively associated with perceived well-being. It was also hypothesized that RAI scores would be positively associated with perceived well-being.

## Materials and Methods

### Sample

An online questionnaire was completed by a sample of 192 Australian residents. Data were collected as a part of a larger study considering musical engagement (see also Krause et al., unpublished); the present research employed only those data concerning individuals who indicated that they were actively participating in a musical activity at the time, such that those individuals who had ceased participating or had never participated in a musical activity are excluded from the present study’s analyses. The present research looks specifically at the variables concerning self-determination theory and perceived well-being, which are not reported in Krause et al., unpublished.

The sample was largely female (63.5% female, 34.9% male, 1.6% declined to respond). Ages ranged from 17 to 85 (*M* = 36.95, *Mdn* = 28.50, *SD* = 19.28); and 52.10% of the sample reported having a university qualification. Regarding the participants’ primary musical activity, 49.7% reported that they played an instrument, 35.1% sung, and 15.2% indicated they were a facilitator (i.e., leading the activity for other people).

Participation in the study was voluntary. Recruitment included the use of online tools, including University student research participation programs, dedicated online study websites, social media postings, and the first author’s/project’s website. Those individuals who participated via a student research participation scheme received course credit.

### Design and Procedure

All participants completed an online questionnaire (using Qualtrics); they were provided with information and consented to participate prior to accessing the questionnaire. Individuals completed the questionnaire as a series of webpages and were thanked and debriefed upon completion.

Individuals stated their age, gender, and country of residence, and were asked to rate the importance of music in their life using a seven-point scale (1 = *not at all important*, 7 = *extremely important*). A direct question asked if the participants were currently participating in a musical activity, had previously participated in a musical activity but were no longer currently participating in a musical activity, or had never participated in a musical activity. After indicating that they were actively participating in a musical activity at the time of completing the questionnaire, participants were asked a series of questions about their current musical participation. In recognition that some individuals may be participating in more than one musical activity, participants were asked to report on their primary activity (e.g., the one concerning which most time is spent) and asked to indicate whether their involvement would be classified as mainly singing, playing an instrument, or facilitating (i.e., leading others in) the activity. Individuals also reported the length of time they had been participating in this particular activity (in years), rated their frequency of involvement using a five-point scale (where 1 = *daily*, 2 = *2–3 times per week*, 3 = *weekly*, 4 = *fortnightly*, 5 = *monthly*), and indicated where the activity took place (by selecting either a domestic setting, community setting, or educational setting). They also estimated the number of other people with whom they regularly participate. While this response was open-ended, the responses were coded as (where 1 = *0*, 2 = *1–5*, 3 = *6–15*, 4 = *16–35*, 5 = *36–75*, and 6 = *76 or more people*).

Participants were asked to complete a Basic Psychological Needs measure concerning music participation. In particular, it was important to address basic psychological needs pertaining to music participation rather than in general. Many self-determination scales have been developed with regard to the context of exercise given the prominence of the theory’s application to this domain (Ryan and Deci, 2007; Hagger and Chatzisarantis, 2008; Wilson et al., 2008); however, music-focused Self-determination scales are scarce. Therefore, an amended version of the Basic Psychological Needs in Exercise Scale (BPNES; Vlachopoulos et al., 2010) was used. Previous research that has employed the BPNES in a variety of domains (e.g., Douglas, 2011; Evans et al., 2013; Niven and Markland, 2016). Following accepted practice, some of the item wording was amended to address musical participation specifically (e.g., ‘I am able to meet the requirements of my music activity’s program,’ ‘My relationships with the people I participate with are close’). Responses were made on a five-point scale (1 = *I don’t agree at* all; 5 = *I completely* agree). Following Vlachopoulos et al.’s (2010) subscale coding, items were averaged in order to compute three scores (one for each of Autonomy, Competency and Relatedness) per participant. The BPNES has demonstrated good reliability and validity (e.g., Vlachopoulos et al., 2010; Lovell et al., 2016; Arrogi et al., 2017). Cronbach’s alpha values were 0.804, 0.855, and 0.831, for autonomy, competency, and relatedness, respectively.

An amended version of the BREQ-2 (Behavioural Regulation in Exercise Questionnaire-2, Markland and Tobin, 2004) which included questions concerning the integrated regulation form (Wilson et al., 2006; McLachlan et al., 2011) was used to measure the quality of each participant’s motivation with regard to their musical activity. Again, in the interest of domain specificity (as previously done, e.g., Niven and Markland, 2016; Teques et al., 2017), amendments to this established, exercise-focused measure were made such that re-phrased items addressed participating in a musical activity specifically. Individuals were asked to respond to the set of 37 items (e.g., ‘I think it is important to make the effort to participate regularly,’ ‘I will feel guilty if I do not participate in my musical activity’) using a five-point scale (0 = *not true for me*; 4 = *very true for me*). Previous research has demonstrated the reliability of this measure (Markland and Tobin, 2004; Lovell et al., 2016; Ntoumanis et al., 2017). Cronbach’s alpha values for the different forms of regulation were as follows: 0.905 for amotivation, 0.821 for external regulation, 0.650 for introjected regulation, 0.775 for identified regulation, 0.854 for integrated regulation, and 0.809 for intrinsic regulation. Adopting the approach used in previous research (e.g., Ryan and Connell, 1989; Niemiec et al., 2006; Wilson et al., 2012; Evans and Bonneville-Roussy, 2016), a RAI score was computed for each participant. To create the RAI score in the present study, the formula employed was: RAI = 3 × Intrinsic + 2 × Integrated + Identified - Introjected - 2 × External – 3 ×Amotivation (Vallerand et al., 2008; Wilson et al., 2012). The participant’s single RAI score was used in subsequent analyses.

Lastly, participants completed Krause et al.’s (2018) measure of the social-psychological well-being benefits of musical participation. Participants responded to the 36 items (e.g., ‘It adds purpose/meaning to my life,’ ‘It does not help me to think about who I am) using a seven-point scale (1 = *Disagree completely*, 7 = *Agree completely*). The total score (for which the 19 negative items were reverse-coded) and five sub-scale scores (addressing the dimensions of mood and coping, esteem and worth, socialization, cognition, and self-actualization respectively) were calculated by averaging the participants’ responses as per Krause et al.’s (2018) sub-scale coding. Cronbach’s alpha values were as follows: 0.951 for the total score, 0.903 for mood and coping, 0.900 for esteem and worth, 0.851 for socializing, 0.771 for cognitive, and 0.747 for self-actualization.

## Results and Discussion

A two-step generalized linear mixed model (GLMM) analysis procedure was used, implemented through SPSS’s (Version 24) GENLINMIXED procedure. In the first step, each of the predictor variables was entered separately with the well-being score as the criterion variable (see Table 1 for the results of the step 1 analyses). The predictor variables were: demographic variables (age, gender, music importance rating, university degree), activity parameters (participation type, length of participation, frequency of participation, location, number of other participants), psychological needs scores (autonomy, competency, relatedness), and RAI score. At step two, the predictor variables that demonstrated a significant relationship with the criterion variable (α < 0.05) were entered together in a single GLMM analysis (α < 0.008).

**Table 1:** Results of the first step of the GLMM analyses concerning well-being score.

Variable	*F*	*DF*	DF_error_	*p*	${\text{η}}_{\text{p}}^{2}$
**Total well-being score**					
Gender	11.025	1	187	0.001	0.056
University degree	7.042	1	189	0.009	0.036
Age	17.504	1	190	<0.001	0.084
Music importance rating (1–7)	31.808	1	188	<0.001	0.145
Activity type	0.981	2	188	0.377	0.010
Length of time participating	8.873	1	186	0.003	0.046
Frequency of participation rating	5.350	1	190	0.022	0.027
Location type	2.285	2	188	0.105	0.024
Number of other participants (grouping)	12.890	1	186	<0.001	0.065
Autonomy score	52.448	1	189	<0.001	0.217
Competency score	84.585	1	187	<0.001	0.311
Relatedness score	36.224	1	187	<0.001	0.162
Relative autonomy index score	160.531	1	173	<0.001	0.481
**Mood and coping well-being score**				
Gender	5.839	1	187	0.017	0.030
University degree	0.738	1	189	0.391	0.004
Age	6.295	1	190	0.013	0.032
Music importance rating (1–7)	25.988	1	188	<0.001	0.121
Activity type	0.862	2	188	0.424	0.009
Length of time participating	6.419	1	186	0.012	0.033
Frequency of participation rating	8.894	1	190	0.003	0.045
Location type	0.074	2	188	0.929	0.001
Number of other participants (grouping)	4.190	1	186	0.042	0.022
Autonomy score	36.935	1	189	<0.001	0.163
Competency score	46.159	1	187	<0.001	0.198
Relatedness score	18.942	1	187	<0.001	0.092
Relative autonomy index score	61.847	1	173	<0.001	0.263
**Esteem and worth well-being score**				
Gender	6.284	1	187	0.013	0.033
University degree	10.900	1	189	0.001	0.055
Age	19.955	1	190	<0.001	0.095
Music importance rating (1–7)	17.985	1	188	<0.001	0.087
Activity type	0.400	2	188	0.671	0.004
Length of time participating	7.914	1	186	0.005	0.041
Frequency of participation rating	5.435	1	190	0.021	0.028
Location type	1.912	2	188	0.151	0.020
Number of other participants (grouping)	6.531	1	186	0.011	0.034
Autonomy score	39.117	1	189	<0.001	0.171
Competency score	61.492	1	187	<0.001	0.247
Relatedness score	18.888	1	187	<0.001	0.092
Relative autonomy index score	90.985	1	173	<0.001	0.345
**Socializing well-being score**					
Gender	12.223	1	187	0.001	0.061
University degree	1.552	1	189	0.214	0.008
Age	9.442	1	190	0.002	0.047
Music importance rating (1–7)	22.109	1	188	<0.001	0.105
Activity type	5.868	2	188	0.003	0.059
Length of time participating	3.037	1	186	0.083	0.016
Frequency of participation rating	0.191	1	190	0.662	0.001
Location type	14.692	2	188	<0.001	0.135
Number of other participants (grouping)	40.123	1	186	<0.001	0.177
Autonomy score	27.146	1	189	<0.001	0.126
Competency score	46.532	1	187	<0.001	0.199
Relatedness score	79.911	1	187	<0.001	0.299
Relative autonomy index score	9.118	1	173	0.003	0.050
**Cognitive well-being score**					
Gender	10.332	1	187	0.002	0.052
University degree	6.200	1	189	0.014	0.032
Age	12.256	1	190	0.001	0.061
Music importance rating (1–7)	19.086	1	188	<0.001	0.092
Activity type	0.254	2	188	0.776	0.003
Length of time participating	6.709	1	186	0.010	0.035
Frequency of participation rating	1.291	1	190	0.257	0.007
Location type	1.853	2	188	0.160	0.019
Number of other participants (grouping)	5.523	1	186	0.020	0.029
Autonomy score	32.452	1	189	<0.001	0.147
Competency score	60.820	1	187	<0.001	0.245
Relatedness score	20.376	1	187	<0.001	0.098
Relative autonomy index score	106.864	1	173	<0.001	0.382
**Self-actualization well-being score**				
Gender	5.891	1	187	0.016	0.031
University degree	12.113	1	189	0.001	0.060
Age	13.781	1	190	<0.001	0.068
Music importance rating (1–7)	18.295	1	188	<0.001	0.089
Activity type	0.122	2	188	0.885	0.001
Length of time participating	4.733	1	186	0.031	0.025
Frequency of participation rating	1.575	1	190	0.211	0.008
Location type	3.698	2	188	0.027	0.038
Number of other participants (grouping)	10.735	1	186	0.001	0.055
Autonomy score	31.551	1	189	<0.001	0.143
Competency score	48.503	1	187	<0.001	0.206
Relatedness score	15.762	1	187	<0.001	0.078
Relative autonomy index score	64.889	1	173	<0.001	0.273

*DF, degrees of freedom.*

This process was repeated in order to conduct six separate analyses, in which each of the total well-being score and five well-being subscale scores served as the respective dependent variable. Tables 2–7 detail the results of these analyses.

**Table 2:** Total well-being score model.

Variable	*F*	*p*	Beta	*t*	95% CI		η^2^
Gender	17.098	<0.001	0.412	4.266	0.215	0.609	0.110
University degree	1.834	0.178	0.140	1.354	-0.064	0.344	0.012
Age	1.891	0.171	0.005	1.375	-0.002	0.011	0.013
Music importance rating (1–7)	4.183	0.043	0.154	2.045	0.005	0.302	0.028
Length of time participating	1.584	0.210	-0.004	-1.259	-0.011	0.002	0.011
Frequency of participation rating	2.058	0.153	0.048	1.435	-0.018	0.114	0.014
Number of other participants (grouping)	0.082	0.775	-0.008	-0.287	-0.064	0.048	0.001
Autonomy score	1.529	0.218	0.252	2.736	0.070	0.434	0.048
Competency score	7.484	0.007	0.252	2.736	0.070	0.434	0.048
Relatedness score	4.791	0.030	0.131	2.189	0.013	0.249	0.032
Relative autonomy index score	84.114	<0.001	0.086	9.171	0.067	0.104	0.364

*Full model: F(11,147) = 32.798, p < 0.001, ${\text{η}}_{\text{p}}^{2}$ = 0.711. Degrees of freedom = 1, 147 for each variable. CI, confidence interval.*

**Table 3:** Mood and coping well-being score model.

Variable	*F*	*p*	Beta	*t*	95% CI		η^2^
Gender	3.871	0.051	0.255	1.968	-0.001	0.511	0.026
Age	0.248	0.619	-0.002	-0.498	-0.009	0.006	0.002
Music importance rating (1–7)	0.218	0.641	0.047	0.467	-0.151	0.245	0.001
Length of time participating	0.109	0.742	-0.001	-0.330	-0.010	0.007	0.001
Frequency of participation rating	0.067	0.797	0.013	0.258	-0.086	0.112	0.000
Number of other participants (grouping)	0.081	0.776	-0.011	-0.285	-0.086	0.064	0.001
Autonomy score	0.097	0.755	0.038	0.312	-0.202	0.277	0.001
Competency score	3.407	0.067	0.208	1.846	-0.015	0.430	0.023
Relatedness score	0.851	0.358	0.063	0.922	-0.072	0.198	0.006
Relative autonomy index score	27.165	<0.001	0.083	5.212	0.051	0.114	0.155

*Full model: F(10,148) = 13.379, p < 0.001, ${\text{η}}_{\text{p}}^{2}$ = 0.475. Degrees of freedom = 1, 148 for each variable. CI, confidence interval.*

**Table 4:** Esteem and worth well-being score model.

Variable	*F*	*p*	Beta	*t*	95% CI		η^2^
Gender	8.941	0.003	0.397	2.990	0.135	0.660	0.057
University degree	0.337	0.563	0.074	0.580	-0.177	0.324	0.002
Age	2.140	0.146	0.006	1.463	-0.002	0.014	0.014
Music importance rating (1–7)	3.003	0.085	0.214	1.733	-0.030	0.458	0.020
Length of time participating	2.022	0.157	-0.006	-1.422	-0.015	0.002	0.014
Frequency of participation rating	1.593	0.209	0.057	1.262	-0.032	0.147	0.011
Number of other participants (grouping)	0.881	0.350	-0.039	-0.938	-0.121	0.043	0.006
Autonomy score	2.001	0.159	-0.179	-1.414	-0.429	0.071	0.013
Competency score	4.550	0.035	0.248	2.133	0.018	0.478	0.030
Relatedness score	0.604	0.438	0.055	0.777	-0.085	0.195	0.004
Relative autonomy index score	44.319	<0.001	0.109	6.657	0.077	0.142	0.232

*Full model: F(11,147) = 12.054, *p* < 0.001, ${\text{η}}_{\text{p}}^{2}$ = 0.474. Degrees of freedom = 1, 147 for each variable. CI, confidence interval.*

**Table 5:** Socializing well-being score model.

Variable	*F*	*p*		Beta	*t*	95% CI		η^2^
Gender	19.001	<0.001		0.559	4.359	0.305	0.812	0.114
Age	0.710	0.401		0.003	0.843	-0.004	0.011	0.005
Music importance rating (1–7)	5.827	0.017		0.278	2.414	0.050	0.506	0.038
Activity type	1.142	0.322	Instrument – Sing	-0.172	-1.218, *p* = 0.225	-0.451	0.107	0.010
			Instrument – Facilitate	0.006	0.044, *p* = 0.965	-0.277	0.289	0.000
			Sing – Facilitate	0.178	1.348, *p* = 0.180	-0.083	0.439	0.012
Location type	2.881	0.059	Domestic setting – Community venue	-0.340	-2.222, *p* = 0.028	-0.642	-0.038	0.032
			Domestic – Educational establishment	-0.126	-0.725, *p* = 0.469	-0.469	0.217	0.004
			Community venue – Educational establishment	0.214	1.532, *p* = 0.128	-0.062	0.490	0.016
Number of other participants (grouping)	1.124	0.291		0.054	1.060	-0.047	0.154	0.008
Autonomy score	0.578	0.448		0.103	0.760	-0.164	0.370	0.004
Competency score	0.363	0.548		0.080	0.602	-0.183	0.344	0.002
Relatedness score	23.557	<0.001		0.487	4.854	0.289	0.686	0.138
Relative autonomy index score	0.079	0.779		0.004	0.281	-0.023	0.031	0.001


*Full model: F(12,147) = 18.307, p < 0.001, ${\text{η}}_{\text{p}}^{2}$ = 0.599. Degrees of freedom = 1, 147 for each variable; except for Activity type and Location type, where Degrees of freedom = 2, 147. CI, confidence interval.*

**Table 6:** Cognitive well-being score model.

Variable	*F*	*p*	Beta	*t*	95% CI		η^2^
Gender	19.749	<0.001	0.585	4.444	0.325	0.845	0.118
University degree	0.479	0.490	0.108	0.692	-0.200	0.415	0.003
Age	1.081	0.300	0.005	1.040	-0.005	0.016	0.007
Music importance rating (1–7)	0.363	0.548	0.060	0.602	-0.136	0.256	0.002
Length of time participating	0.021	0.884	-0.001	-0.146	-0.010	0.009	0.000
Number of other participants (grouping)	2.037	0.156	-0.061	-1.427	-0.409	0.088	0.014
Autonomy score	1.624	0.205	-0.160	-1.274	-0.409	0.088	0.011
Competency score	5.692	0.018	0.284	2.386	0.049	0.519	0.037
Relatedness score	2.906	0.090	0.147	1.705	-0.023	0.317	0.019
Relative autonomy index score	55.428	<0.001	0.100	7.445	0.074	0.127	0.272

*Full model: F(10,148) = 20.583, p < 0.001, ${\text{η}}_{\text{p}}^{2}$ = 0.582. Degrees of freedom = 1, 148 for each variable. CI, confidence interval.*

**Table 7:** Self-actualization well-being score model.

Variable	*F*	*p*		Beta	*t*	95% CI		η^2^
Gender	6.645	0.011		0.414	2.578	0.097	0.731	0.044
University degree	0.382	0.537		-0.101	-0.618	-0.424	0.222	0.003
Age	0.509	0.477		0.003	0.714	-0.006	0.012	0.004
Music importance rating (1–7)	1.271	0.261		0.159	1.127	-0.120	0.438	0.009
Length of time participating	1.939	0.166		-0.008	-1.392	-0.018	0.003	0.013
Location type	0.184	0.832	Domestic setting – Community venue	-0.035	-0.180, *p* = 0.857	-0.422	0.351	0.000
			Domestic – Educational establishment	-0.119	-0.552, *p* = 0.582	-0.545	0.307	0.002
			Community venue – Educational establishment	-0.084	-0.495, *p* = 0.621	-0.418	0.250	0.002
Number of other participants (grouping)	0.002	0.966		-0.002	-0.043	-0.117	0.112	0.000
Autonomy score	1.427	0.234		-0.171	-1.195	-0.453	0.112	0.010
Competency score	2.143	0.145		0.234	1.464	-0.082	0.550	0.015
Relatedness score	1.169	0.281		0.116	1.081	-0.096	0.329	0.008
Relative autonomy index score	20.921	<0.001		0.097	4.574	0.055	0.139	0.126

*Full model: F(12,145) = 11.897, *p* < 0.001, ${\text{η}}_{\text{p}}^{2}$ = 0.496. Degrees of freedom = 12, 145 for each variable; except for Location type, where Degrees of freedom = 2, 145. CI, confidence interval.*

**FIGURE 1:**
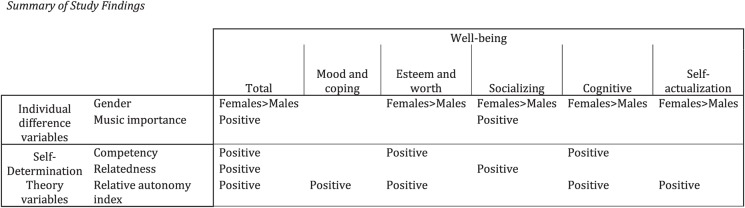
Summary of study findings.

As evident in Tables 2–7 and summarized in Figure 1, the individual models displayed similar patterns of results across the set of analyses. In particular, with regard to gender, the results indicate that females reported experiencing greater perceived well-being benefits on five of the six measures than males (all except for the mood and coping sub-scale score). The music importance rating was positively associated with the total well-being score and socializing sub-scale sore. Indeed, the evidence for positive associations between musical participation and perceived social well-being is growing (e.g., von Lob et al., 2010; Jutras, 2011; Rohwer and Rohwer, 2012; Creech et al., 2013b; McQueen et al., 2013; Krause et al., 2018).

The RAI score demonstrated a significant, positive association in five of the six analyses (all except for the socializing well-being score). More simply, greater well-being was associated with internalized motivation to participate in music (see Figure 1). Indeed, when the RAI score demonstrated a significant association, it accounted for the largest percentage of variance in each analysis, suggesting the particular importance of self-regulated motivation. The strong, positive associations between internalized motivation and perceived well-being support Self-Determination Theory’s links between autonomous motivation and well-being (e.g., Deci and Ryan, 2000).

As seen in Figure 1, with regard to the three basic psychological needs, competency (defined as the need to be effective in one’s efforts) was positively associated with the total, esteem and self-worth, and cognitive well-being sub-scale scores. Relatedness (the need to be socially connected) was positively associated with the total and socializing well-being sub-scale score. These findings demonstrate logical associations between those two types of psychological needs and the well-being types: while obviously not indicative of a causal effect, these findings indicate, within the context of specifically musical participation, a relationship between feeling related to other people and experiencing social well-being benefits, as has been reported in much research (e.g., von Lob et al., 2010; Jutras, 2011; Rohwer and Rohwer, 2012; Creech et al., 2013b; McQueen et al., 2013; Krause et al., 2018); and the positive relationship between competency and various aspects of both social and cognitive well-being supports prior research that also demonstrates links between musical participation and cognitive well-being (e.g., Gick, 2011; Kokotsaki and Hallam, 2011; Creech et al., 2013a). Indeed, relatedness was particularly important in Quested et al.’s (2018) community gardening investigation, as well as Sebire et al.’s (2016) dance research. Autonomy (the need to feel that one’s activities are self-endorsed and volitional) did not demonstrate any significant associations with well-being: the absence of significant findings concerning autonomy *per se* is interesting, given that previous research findings have linked autonomy to motivation and engagement (e.g., Williams and Deci, 1998; Reeve et al., 2004; Valenzuela et al., 2018), as well as well-being (e.g., Ilardi et al., 1993).

Additionally, in the full models (Tables 2–7), the specific nature of musical participation (i.e., instrument versus singing versus facilitation) was not associated with well-being. It is also slightly surprising, but nonetheless encouraging, that well-being correlates of musical participation were not related to length of time and frequency of participating when considering overt measures of competence, relatedness, and autonomy as well. The lack of any such associations can be interpreted positively: experiencing well-being benefits in the context of musical participation does not appear to hinge on the particulars of the musical activity itself. Rather, people can select musical activities aligned with their personal preferences and which fit within their lifestyles without implications for their probability of experiencing greater well-being. Of course, it would not be fruitful to simply force people to participate in musical activities (e.g., at school, via private lessons, or in community spaces) without striving to also increase feelings of competence, relatedness, and autonomy. Our results suggest that self-determination is considerably more important than simple attendance. Similarly, the only demographic variable related to well-being was gender, so that age and education level are also unrelated to the relationship between musical participation and well-being. Thus, these findings have broad implications for music educators, community musicians, music therapists, and others who are involved in facilitating participation opportunities.

In particular, rather than the activity itself or individual differences (such as age and education level), the present findings suggest that it is competence, relatedness, and autonomy (expressed in terms of the RAI) that predict well-being in the context of musical participation, as predicted by self-determination theory. Indeed, the present findings (as summarized in Figure 1) suggest that musical participation opportunities should be interesting, challenging, and offered in contexts that support autonomous motivation. Clearly then, the challenge for music educators, community musicians, and music therapists that arises from these results concerns specifically how they might foster self-perceptions of competence, relatedness, and autonomy via a number of specific and more general approaches to musical participation.

Some of the arguments here also suggest interesting directions for future research. The role of the facilitator (i.e., the person who leads the activity) was not examined here, which is particularly unfortunate given that he/she may have an important role to play in shaping competence, relatedness, and autonomy. In the context of specifically musical participation, and particularly music education, the role of the facilitator is clearly crucial (e.g., Delano and Royse, 1987; Corenblum and Marshall, 1998; Evans et al., 2013). There exists, of course, an extensive literature from outside music that specifically addresses the form that these approaches might take (e.g., Gagné and Deci, 2005; Jang et al., 2010; Hagger et al., 2015). Facilitators can draw on the findings indicating how, in educational settings, autonomy supportive teachers positively influence students’ psychological needs, motivation, and engagement (e.g., Niemiec and Ryan, 2009; Jang et al., 2010; Hagger et al., 2015). Specific to music education, Evans (2015) offered suggestions as to pedagogical strategies to promote the fulfillment of the psychological needs. It will be important for future research to empirically consider which particular pedagogical techniques are perceived by students as needs-supporting and needs-thwarting.

Further, longitudinal research could also consider these ideas with emphasis on continuation versus cessation of participation. For example, such work might consider associations between the fulfillment of psychological needs and the issues surrounding continued participation, such as potential barriers. It may be that musical participation occupies varying levels of importance at different points in someone’s life. Participation must ‘fit’ within a person’s lifestyle: examining the issues related to age and life-stage may illuminate how some people are able to prioritize/continue/cease their musical participation.

Additionally, while Self-Determination Theory has been applied to many phenomena involving motivation across multiple domains, its application to musical behaviors is nascent. Thus, the present research makes a novel contribution to knowledge by providing empirical evidence that supports the application of Self-Determination Theory to explain musical participation. However, the exploratory nature of this research should be noted. Thus, additional research is needed to refine its application as well as further explore particular aspects of well-being. For instance, future research could consider specifically eudemonic well-being with regard to the motivations and functions of continued participation (Groarke and Hogan, 2016).

In summary, the present study used Self-Determination Theory to examine musical participation and well-being. The pattern of results reiterates the positive associations between musical participation and benefits to one’s emotional and social well-being; and makes clear that that feeling competent, a sense of relatedness to others, and autonomous motivation should be prioritized in music making opportunities. The findings indicate that Self-Determination Theory (including the mini theories of Basic Psychological Needs and Organismic Integration Theory in particular) offers a useful theoretical framework to understanding musical participation with regard to well-being.

## Data Availability

The datasets for this study will not be made publicly available because the ethics permissions require that the data be kept and destroyed.

## Ethics Statement

This project received ethical approval from Curtin University.

## Author Contributions

AK, JD, and AN jointly developed the conceptual and methodological approach and co-wrote the discussion. AK oversaw the ethics, participant recruitment, data collection, and developed the literature review and sketched the shape of the final article. AK and AN undertook the data analysis.

## Conflict of Interest Statement

The authors declare that the research was conducted in the absence of any commercial or financial relationships that could be construed as a potential conflict of interest.

## References

[B1] Alivernini F. Lucidi F. (2011). Relationship between social context, self-efficacy, motivation, academic achievement, and intention to drop out of high school: a longitudinal study. *J. Educ. Res.* 104 241–252. 10.1080/00220671003728062

[B2] Arrogi A. Schotte A. Bogaerts A. Boen F. Seghers J. (2017). Short- and long-term effectiveness of a three-month individualized need- supportive physical activity counseling intervention at the workplace. *BMC Publ. Health* 17:52. 10.1186/s12889-016-3965-1 28069016 PMC5223544

[B3] Baard P. P. Deci E. L. Ryan R. M. (2004). Intrinsic need satisfaction: a motivational basis of performance and well-being in two work settings. *J. Appl. Soc. Psychol.* 34 2045–2068. 10.1111/j.1559-1816.2004.tb02690.x

[B4] Black A. E. Deci E. L. (2000). The effects of instructors’ autonomy support and students’ autonomous motivation on learning organic chemistry: a self-determination theory perspective. *Sci. Educ.* 84 740–756. 10.1002/1098-237X(200011)84:6<740::AID-SCE4>3.0.CO;2-3

[B5] Bonneville-Roussy A. Evans P. Verner-Filion J. Vallerand R. J. Bouffard T. (2017). Motivation and coping with the stress of assessment: gender differences in outcomes for university students. *Contemp. Educ. Psychol.* 48 28–42. 10.1016/j.cedpsych.2016.08.003

[B6] Bonneville-Roussy A. Vallerand R. J. Bouffard T. (2013). The roles of autonomy support and harmonious and obsessive passions in educational persistence. *Learn. Individ. Diff.* 24 22–31. 10.1016/j.lindif.2012.12.015

[B7] Chatzisarantis N. L. D. Hagger M. S. (2009). Effects of an intervention based on self-determination theory on self-reported leisure-time physical activity participation. *Psychol. Health* 24 29–48. 10.1080/08870440701809533 20186638

[B8] Clift S. Hancox G. (2010). The significance of choral singing for sustaining psychological wellbeing: Findings from a survey of choristers in england. Australia and Germany. *Music Perform. Res.* 3 79–96.

[B9] Clift S. Hancox G. Staricoff R. Whitmore C. (2008). *Singing and Health: Summary of A Systematic Mapping and Review of Non-Clinical Research*. Canterbury: Canterbury Christ Church University.

[B10] Coleman D. Iso-Ahola S. E. (1993). Leisure and health: The role of social support and self-determination. *J. Leis. Res.* 25 111–128. 10.1080/00222216.1993.11969913

[B11] Corenblum B. Marshall E. (1998). The band played on: predicting students’ intentions to continue studying music. *J. Res. Music Educ.* 46 128–140. 10.2307/3345765

[B12] Countryman J. (2014). Missteps, flaws, and morphings in children’s musical play: snapshots from school playgrounds. *Res. Stud. Music Educ.* 36 3–18. 10.1177/1321103X14528456

[B13] Creech A. Hallam S. Gaunt H. McQueen H. Pincas A. Varvarigou M. (2013a). The power of music in the lives of older adults. *Res. Stud. Music Educ.* 35 87–102. 10.1177/1321103X13478862

[B14] Creech A. Hallam S. Varvarigou M. McQueen H. Gaunt H. (2013b). Active music making: a route to enhanced subjective well-being among older people. *Pers. Publ. Health* 133 36–43. 10.1177/1757913912466950 23308006

[B15] Deci E. L. Ryan R. M. (1985a). *Intrinsic Motivation and Self-Determination in Human Behavior: Perspectives in Social Psychology*. New York, NY: Plenum 10.1007/978-1-4899-2271-7

[B16] Deci E. L. Ryan R. M. (1985b). The general causality orientations scale: self-determination in personality. *J. Res. Pers.* 19 109–134. 10.1016/0092-6566(85)90023-6

[B17] Deci E. L. Ryan R. M. (1987). The support of autonomy and the control of behavior. *J. Pers. Soc. Psychol.* 53 1024–1037. 10.1037/0022-3514.53.6.1024 3320334

[B18] Deci E. L. Ryan R. M. (1991). “A motivational approach to self: Integration in personality,” in *Current Theory and Research in Motivation, Nebraska Symposium on Motivation, 1990: Perspectives on Motivation*, ed. Dienstbier R. A. (Lincoln, NE: University of Nebraska Press). 2130258

[B19] Deci E. L. Ryan R. M. (2000). The “what” and “why” of goal pursuits: human needs the self-determination of behavior. *Psychol. Inq.* 11 227–268. 10.1207/S15327965PLI1104_01 27055568

[B20] Deci E. L. Ryan R. M. (2008). Facilitating optimal motivation and psychological well-being across life’s domains. *Can. Psychol.* 49 14–23. 10.1037/0708-5591.49.1.14

[B21] Deci E. L. Ryan R. M. Gagné M. Leone D. R. Usunov J. Kornazheva B. P. (2001). Need satisfaction, motivation, and well-being in the work organizations of a former eastern bloc country. *Pers. Soc. Psychol. Bull.* 27 930–942. 10.1177/0146167201278002

[B22] Delano A. Royse D. (1987). Factors influencing the decision of college freshmen to participate or not to participate in kent state university music ensembles. *Contrib. Music Educ.* 14 9–18.

[B23] Douglas K. A. (2011). *A Descriptive Analysis of The Psychological Needs of Adults Participating in Music Ensembles: A Survey of the New Horizons International Music Association Ensemble Participants* (Doctoral Dissertation) Greensboro: The University of North Carolina.

[B24] Evans P. (2015). Self-determination theory: An approach to motivation in music education. *Music Sci.* 19 65–83. 10.1177/1029864914568044

[B25] Evans P. Bonneville-Roussy A. (2016). Self-determined motivation for practice in university music students. *Psychol. Music* 44 1095–1110. 10.1177/0305735615610926

[B26] Evans P. McPherson G. E. Davidson J. W. (2013). The role of psychological needs music in ceasing activities music learning. *Psychol. Music* 41 600–619. 10.1177/0305735612441736

[B27] Ferrand C. Martinent G. Durmaz N. (2014). Psychological need satisfaction and well-being in adults aged 80 years and older living in residential homes: using a self-determination theory perspective. *J. Aging Stud.* 30 104–111. 10.1016/j.jaging.2014.04.004 24984913

[B28] Freer E. Evans P. (2018). Psychological needs satisfaction and value in students’ intentions to study music in high school. *Psychol. Music* 46 881–895. 10.1177/0305735617731613

[B29] Gagné M. Deci E. L. (2005). Self-determination theory and work motivation. *J. Organ. Behav.* 26 331–362. 10.1002/job.322

[B30] Georgiadis M. M. Biddle S. J. H. Stavrou N. A. (2006). Motivation for weight-loss diets: a clustering, longitudinal field study using self-esteem and self-determination theory perspectives. *Health Educ. J.* 65 53–72. 10.1177/0017896906066067

[B31] Gick M. L. (2011). Singing, health and well being: a health psychologist’s review. *Psychomusicology* 21 176–207. 10.1037/h0094011

[B32] Groarke J. M. Hogan M. J. (2016). Enhancing wellbeing: an emerging model of the adaptive functions of music listening. *Psychol. Music* 44 769–791. 10.1177/0305735615591844

[B33] Hagger M. S. Chatzisarantis N. L. D. (2008). Self-determination theory and the psychology of exercise. *Int. Rev. Sport Exer. Psychol.* 1 79–103. 10.1080/17509840701827437

[B34] Hagger M. S. Chatzisarantis N. L. D. Harris J. (2006). From psychological need satisfaction to intentional behavior: testing a motivational sequence in two behavioral contexts. *PSPB* 32 131–148. 10.1177/0146167205279905 16382077

[B35] Hagger M. S. Sultan S. Hardcastle S. J. Chatzisarantis N. L. D. (2015). Perceived autonomy support and autonomous motivation toward mathematics activities in educational and out-of-school contexts is related to mathematics homework behavior and attainment. *Contemp. Educ. Psychol.* 41 111–123. 10.1016/j.cedpsych.2014.12.002

[B36] Ilardi B. C. Leone D. Kasser T. Ryan R. M. (1993). Employee and supervisor ratings of motivation: main effects and discrepancies associated with job satisfaction and adjustment in a factory setting. *J. Appl. Soc. Psychol.* 23 1789–1805. 10.1111/j.1559-1816.1993.tb01066.x

[B37] Jang H. Kim E. J. Reeve J. (2012). Longitudinal test of self-determination theory’s motivation mediation model in a naturally occurring classroom context. *J. Educ. Psychol.* 104 1175–1188. 10.1037/a0028089

[B38] Jang H. Reeve J. Deci E. L. (2010). Engaging students in learning activities: It is not autonomy support or structure, but autonomy support and structure. *J. Educ. Psychol.* 102 588–600. 10.1037/a0019682

[B39] Jutras P. J. (2011). The benefits of new horizons band participation as self-reported by selected new horizons band members. *Bull. Council Res. Music Educ.* 187 65–84.

[B40] Kokotsaki D. Hallam S. (2011). The perceived benefits of participative music making for non-music university students: a comparison with music students. *Music Educ. Res.* 13 149–172. 10.1080/14613808.2011.577768

[B41] Krause A. E. Davidson J. W. (2018). Effective educational strategies to promote life-long musical investment: perceptions of educators. *Front. Psychol.* 9:1977. 10.3389/fpsyg.2018.01977 30410455 PMC6209638

[B42] Krause A. E. Davidson J. W. North A. C. (2018). Musical activity and well-being: a new quantitative measurement instrument. *Music Perc.* 35 454–474. 10.1525/mp.2018.35.4.454

[B43] Küpers E. van Dijk M. McPherson G. E. van Geert P. (2014). A dynamic model that links skill acquisition with self-determination in instrumental music lessons. *Music. Sci.* 18 17–34. 10.1177/1029864913499181

[B44] Kuykendall L. Tay L. Ng V. (2015). Leisure engagement and subjective well-being: a meta-analysis. *Psychol. Bull.* 141 364–403. 10.1037/a0038508 25602273

[B45] Laukka P. (2007). Uses of music and psychological well-being among the elderly. *J. Happ. Stud.* 8 215–341. 10.1007/s10902-006-9024-3

[B46] Lee J. Davidson J. W. McFerran K. S. (2016). Registered music therapists’ motivations and perceptions of the impact of their practices on the well-being of clients and themselves. *Aus. J. Music Ther.* 27 27–43.

[B47] Lombas A. S. Esteban M. Á (2018). The confounding role of basic needs satisfaction between self-determined motivation and well-being. *J. Happ. Stud.* 19 1305–1327. 10.1007/s10902-017-9874-x

[B48] Lovell G. P. Gordon J. A. R. Mueller M. B. Mulgrew K. Sharman R. (2016). Satisfaction of basic psychological needs, self-determined exercise motivation, and psychological well-being in mothers exercising in group-based versus individual-based contexts. *Health Care Women Int.* 37 568–582. 10.1080/07399332.2015.1078333 26252897

[B49] MacDonald R. A. R. (2013). Music, health, and well-being: A review. *Int. J. Qual. Stud. Health Well-Being* 8:20635. 10.3402/qhw.v8i0.20635 23930991 PMC3740599

[B50] MacDonald R. A. R. Kreutz G. Mitchell L. A. (2012). “What is music, health, and wellbeing and why is it important?,” in *Music, Health, and Wellbeing*, eds MacDonald R. A. R. Kreutz G. Mitchell L. A. (Oxford, UK: Oxford University Press), 3–11.

[B51] MacIntyre P. D. Schnare B. Ross J. (2018). Self-determination theory and motivation for music. *Psychol. Music* 46 699–715. 10.1177/0305735617721637

[B52] Markland D. Tobin V. (2004). A modification to the behavioural regulation in exercise questionnaire to include an assessment of amotivation. *J. Sport Exer. Psychol.* 26 191–196. 10.1123/jsep.26.2.191

[B53] McLachlan S. Spray C. Hagger M. S. (2011). The development of a scale measuring integrated regulation in exercise. *Br. J. Health Psychol.* 16 722–743. 10.1348/2044-8287.002009 21199546

[B54] McPherson G. E. O’Neill S. A. (2016). “Musical potential,” in *The Oxford Handbook of Music Psychology*, eds Hallam S. Cross I. Thaut M. (Oxford, UK: Oxford University Press), 433–448.

[B55] McQueen H. Hallam S. Creech A. Varvarigou M. (2013). A philosophical perspective on leading music activities for the over 50s. *Int. J. Lifelong Educ.* 32 353–377. 10.1080/02601370.2012.738432

[B56] Milyavskaya M. Koestner R. (2011). Psychological needs, motivation, and well-being: a test of self-determination theory across multiple domains. *Pers. Individ. Diff.* 50 387–391. 10.1016/j.paid.2010.10.029

[B57] Niemiec C. P. Lynch M. F. Vansteenkiste M. Bernstein J. Deci E. L. Ryan R. M. (2006). The antecedents and consequences of autonomous self-regulation for college: a self-determination theory perspective on socialization. *J. Adolesc.* 29 761–775. 10.1016/j.adolescence.2005.11.009 16412502

[B58] Niemiec C. P. Ryan R. M. (2009). Autonomy, competence, and relatedness in the classroom: applying self-determination theory to educational practice. *Theory Res. Educ.* 7 133–144. 10.1177/1477878509104318

[B59] Niven A. G. Markland D. (2016). Using self-determination theory to understand motivation for walking: instrument development and model testing using Bayesian structural equation modeling. *Psychol. Sport Exer.* 23 90–100. 10.1016/j.psychsport.2015.11.004

[B60] Ntoumanis N. Thøgersen-Ntoumani C. Quested E. Hancox J. (2017). The effects of training group exercise class instructors to adopt a motivationally adaptive communication style. *Scand. J. Med. Sci. Sports* 27 1026–1034. 10.1111/sms.12713 27283879

[B61] Oga-Baldwin W. L. Q. Nakata Y. Parker P. Ryan R. M. (2017). Motivating young language learners: a longitudinal model of self-determined motivation in elementary school foreign language classes. *Contemp. Educ. Psychol.* 49 140–150. 10.1016/j.cedpsych.2017.01.010

[B62] O’Neill S. A. McPherson G. E. (2002). “Motivation,” in *The Science & Psychology of Music Performance: Creative Strategies for Teaching and Learning*, eds Parncutt R. McPherson G. E. (Oxford, UK: Oxford University Press), 31–46. 10.1093/acprof:oso/9780195138108.003.0003

[B63] Patrick H. Canevello A. Knee C. R. Lonsbary C. (2007). The role of need fulfillment in relationship functioning and well-being: a self-determination theory perspective. *J. Pers. Soc. Psychol.* 92 434–457. 10.1037/0022-3514.92.3.434 17352602

[B64] Quested E. Thøgersen-Ntoumani C. Uren H. Hardcastle S. J. Ryan R. M. (2018). Community gardening: basic psychological needs individual as mechanisms to enhance well community-being. *Ecopsychology* 10 173–180. 10.1089/eco.2018.0002

[B65] Reeve J. Jang H. Carrell D. Jeon S. Barch J. (2004). Enhancing students’ engagement by increasing teachers’ autonomy support. *Motiv. Emot.* 28 147–169. 10.1023/B:MOEM.0000032312.95499.6f

[B66] Reinboth M. Duda J. L. (2006). Perceived motivational climate, need satisfaction and indices of well-being in team sports: a longitudinal perspective. *Psychol. Sport Exer.* 7 269–286. 10.1016/j.psychsport.2005.06.002

[B67] Roberts J. C. (2018). Self-determination theory and children’s singing games in and out of the classroom: a literature review. *Update* 36 12–19. 10.1177/8755123317741488

[B68] Rohwer D. Rohwer M. (2012). How participants envision community music in welsh men’s choirs. *Res. Issues Music Educ.* 10:3 Available at: http://ir.stthomas.edu/rime/vol10/iss1/3

[B69] Ryan R. M. Connell J. P. (1989). Perceived locus of causality and internalization: examining reasons for acting in two domains. *J. Pers. Soc. Psychol.* 57 749–761. 10.1037/0022-3514.57.5.749 2810024

[B70] Ryan R. M. Deci E. L. (2000). Self-determination theory and the facilitation of intrinsic motivation, social development, and well-being. *Am. Psychol.* 55 68–78. 10.1037/0003-066X.55.1.68 11392867

[B71] Ryan R. M. Deci E. L. (2002). “An overview of Self-determination Theory: An organismic-dialectical perspective,” in *Handbook of Self-Determination Research*, eds Deci E. L. Ryan R. M. (Rochester, NY: The University of Rochester Press).

[B72] Ryan R. M. Deci E. L. (2007). “Active human nature: Self-determination theory and the promotion and maintenance of sport, exercise, and health,” in *Intrinsic Motivation And Self-Determination in Exercise and Sport*, eds Hagger M. S. Chatzisarantis N. L. D. (Champaign, IL: Human Kinetics), 1–20.

[B73] Schatt M. D. (2018). Middle school band students’ self-determination to practice. *Psychol. Music* 46 208–221. 10.1177/0305735617705008

[B74] Sebire S. J. Kesten J. M. Edwards M. J. May T. Banfield K. Tomkinson K. (2016). Using self-determination theory to promote adolescent girls’ physical activity: exploring the theoretical fidelity of the bristol girls dance project. *Psychol. Sport Exer.* 24 100–110. 10.1016/j.psychsport.2016.01.009 27175102 PMC4852534

[B75] Seymour G. Peterman A. (2018). Context and measurement: an analysis of the relationship between intrahousehold decision making and autonomy. *World Dev.* 111 97–112. 10.1016/j.worlddev.2018.06.027

[B76] Sheldon K. M. Krieger L. S. (2007). Understanding the negative effects of legal education on law students: a longitudinal test of self-determination theory. *Pers. Soc. Psychol. Bull.* 33 883–897. 10.1177/0146167207301014 17483395

[B77] Skingley A. Bungay H. Clift S. (2011). Researching participatory arts, well-being and health: some methodological issues. *J. Arts Commun.* 3 73–87. 10.1386/jaac.3.1.73_1 27610359

[B78] Soenens B. Sierens E. Vansteenkiste M. Dochy F. Goossens L. (2012). Psychologically controlling teaching: Examining outcomes, antecedents, and mediators. *J. Educ. Psychol.* 104 108–120. 10.1037/a0025742

[B79] Teques P. Calmeiro L. Silva C. Borrego C. (2017). Validation and adaptation of the physical activity enjoyment scale (PACES) in fitness group exercisers. *J. Sport Health Sci.* 1–6. 10.1016/j.jshs.2017.09.010 PMC7411091 32768128

[B80] Ulstad S. O. Halvari H. Deci E. L. (2018). The role of students’ and teachers’ ratings of autonomous motivation in a self-determination theory model predicting participation in physical education. *Scand. J. Educ. Res.* 1–16. 10.1080/00313831.2018.1476917

[B81] Valenzuela R. Codina N. Pestana J. V. (2018). Self-determination theory applied to flow in conservatoire music practice: the roles of perceived autonomy and competence, and autonomous and controlled motivation. *Psychol. Music* 46 33–48. 10.1177/0305735617694502

[B82] Vallerand R. J. Pelletier L. G. Koestner R. (2008). Reflections on self-determination theory. *Can. Psychol.* 49 257–262. 10.1037/a0012804

[B83] Van den Broeck A. Ferris D. L. Chang C.-H. Rosen C. C. (2016). A review of self-determination theory’s basic psychological needs at work. *J. Manag.* 42 1195–1229. 10.1177/0149206316632058

[B84] Vansteenkiste M. Simons J. Lens W. Sheldon K. M. Deci E. L. (2004). Motivating learning, performance, and persistence: the synergistic effects of intrinsic goal contents and autonomy-supportive contexts. *J. Pers. Soc. Psychol.* 87 246–260. 10.1037/0022-3514.87.2.246 15301630

[B85] Vlachopoulos S. P. Ntoumanis N. Smith A. L. (2010). The basic psychological needs in exercise scale: translation and evidence for cross-cultural validity. *Int. J. Sport Exer. Psychol.* 8 394–412. 10.1080/1612197X.2010.9671960 20977048

[B86] von Lob G. Camic P. Clift S. (2010). The use of singing in a group as a response to adverse life events. *Int. J. Ment. Health Prom.* 12 45–53. 10.1080/14623730.2010.9721818 30487379

[B87] Williams G. C. Deci E. L. (1998). The importance of supporting autonomy in medical education. *Ann. Inter. Med.* 129 303–308. 10.7326/0003-4819-129-4-199808150-00007 9729184

[B88] Wilson P. M. Mack D. E. Grattan K. P. (2008). Understanding motivation for exercise: a self-determination theory perspective. *Can. Psychol.* 49 250–256. 10.1037/a0012762

[B89] Wilson P. M. Rodgers W. M. Loitz C. C. Scime G. (2006). It’s who i am really! the importance of integrated regulation in exercise contexts. *J. Appl. Biobehav. Res.* 11 79–104. 10.1111/j.1751-9861.2006.tb00021.x

[B90] Wilson P. M. Sabiston C. M. Mack D. E. Blanchard C. M. (2012). On the nature and function of scoring protocols used in exercise motivation research: an empirical study of the behavioral regulation in exercise questionnaire. *Psychol. Sport Exer.* 13 614–622. 10.1016/j.psychsport.2012.03.009

